# The Use of Probiotics for Management and Improvement of Reproductive Eubiosis and Function

**DOI:** 10.3390/nu14040902

**Published:** 2022-02-21

**Authors:** Nesrein M. Hashem, Antonio Gonzalez-Bulnes

**Affiliations:** 1 Department of Animal and Fish Production, Faculty of Agriculture (El-Shatby), Alexandria University, Alexandria 21545, Egypt; 2 Departamento de Produccion y Sanidad Animal, Facultad de Veterinaria, Universidad Cardenal Herrera-CEU, CEU Universities, C/Tirant lo Blanc, 7, Alfara del Patriarca, 46115 Valencia, Spain

**Keywords:** fertility, mammals, eubiosis, probiotics

## Abstract

Reproductive tract dysbiosis, due to the action of pathogens and/or unhealthy lifestyle, has been related to many reproductive diseases and disorders in mammalian species. Classically, such a problem has been confronted by the administration of antibiotics. Despite their effectiveness for controlling disease, treatments with antibiotics may negatively affect the fertility of males and females and, mainly, may induce antibiotic resistance. Accordingly, safer alternatives for maintaining reproductive system eubiosis, such as probiotics, are required. The present review summarizes the current knowledge on the biodiversity of the microbiota at the reproductive tract, possible changes in the case of dysbiosis, and their relationships with adequate reproductive health and functioning in both females and males. Afterwards, mechanisms of action and benefits of different probiotics are weighed since the biological activities of probiotics may provide a promising alternative to antibiotics for maintaining and restoring reproductive eubiosis and function. However, at present, it is still necessary for further research to focus on: (a) identifying mechanisms by which probiotics can affect reproductive processes; (b) the safety of probiotics to the host, specifically when consumed during sensitive reproductive windows such as pregnancy; and (c) the hazards instructions and regulatory rules required for marketing these biological-based therapies with sufficient safety. Thus, in this review, to draw a comprehensive overview with a relatively low number of clinical studies in this field, we showed the findings of studies performed either on human or animal models. This review strategy may help provide concrete facts on the eligible probiotic strains, probiotics colonization and transfer route, and prophylactic and/or therapeutic effects of different probiotic strains.

## 1. Introduction

Plenty of unfriendly factors can adversely affect the community of the reproductive tract’s microbiome in both human beings and/or animals because of either modern lifestyle or industrialization and intensification of production, leading to reproductive tract dysbiosis and the need for restoring a eubiotic state.

Reproductive dysbiosis has been related to several reproductive disorders and infertility. The use of antibiotics and the use of probiotics are basically two hypothesized approaches that could be used to control reproductive tract microbiota. In this context, several studies have confirmed the beneficial effects of antibiotics to tackle many reproductive microbial infectious diseases and related infertility [1,2,3]. On the other hand, treatment with antibiotics, even if for the short-term, can negatively affect the fertility of males and females [3,4]. Furthermore, the widespread use of antibiotics poses a serious health risk due to the development and spread of multiple-antibiotic-resistant microbial species [5]. These aspects evoke scientists to find more safe and ecofriendly alternatives for maintaining reproductive system eubiosis, such as probiotics. Etymologically, the term probiotic includes two clauses: The first clause is originated from the Latin preposition *pro* that means “for” or “in support”, and the second clause is originated from the Greek adjective *biotic* that means “life”, so together, they mean “in support of life”. Scientifically, probiotics refer to viable microorganisms that have beneficial effects on the consumers’ health. 

Many studies have shown the positive effects of probiotics-based therapies on reproductive health in human [6,7,8,9] and animal models [10,11]. Probiotics support reproductive tract eubiosis and the fertility of the host due to their antimicrobial, antioxidant, anti-inflammatory, and immunomodulatory activities. In the field of human reproductive health, the use of probiotics either through oral or vaginal route of administration has proved its effectiveness against many reproductive disorders in women (i.e., vaginosis, polycystic ovary syndrome, and pre-term delivery [12,13]). Similarly, probiotics show positive effects on male fertility and semen quality [8]. In animal models, probiotics have been used to mitigate the negative effects of many reproductive disorders, specifically those impairing the immune-inflammatory system and/or due to the action of infectious diseases such as post-partum endometritis [14]. 

The present review sheds the light on: 1- the relationships between reproductive tract eubiosis/dysbiosis and reproductive health and functioning; 2- the use of probiotics for maintaining and restoring reproductive eubiosis and function with emphasis on their properties, mechanisms of action, and benefits for reproductive function; 3- the safety of probiotics to the host; and 4- the litigation and regulatory rules required for marketing these biological-based therapies.

## 2. Biodiversity of the Reproductive Tract Microbiota and Fertility

### 2.1. Human Model

A direct relationship between the biodiversity of reproductive tract microbiota and reproductive health and the completion of different reproductive events has been confirmed. The microbial biodiversity of the reproductive tract is highly dynamic and is affected by several internal and/or external factors. However, for maintaining adequate reproductive functioning and health, the prevalence of beneficial microbes should be ensured. The prevalence of some microbial species has been related to sufficient reproductive health and functions. Some microbial species are classified as beneficial microbial strains, mainly *Lactobacillus* species, as well as *Acinetobacter*, *Pseudomonas*, *Comamonadaceae*, *Jonquetella*, *Fusobacterium*, *Bacteroidetes*, and *Preovotella* [15]. In contrast, other microbial species such as *Escherichia coli*, *Trueperella pyogenes, Enterococci*, *Enterobacteriaceae*, *Streptococci*, *Ureaplasma*, and *Staphylococci* are classified as pathogenic microbes and have been linked to several reproductive disorders [1,16,17].

Either in males or females, reproductive tract dysbiosis has been related to many reproductive dysfunction and infertility. Dysbiosis (the dominance of pathogen-driven microbes) of the reproductive tract can disrupt numerous vital reproductive functions such as gametogenesis, steroidogenesis, embryo development and implantation, and establishment of pregnancy [18]. 

In women, many gynecological diseases are related to the dysbiosis of endometrial microbiome, i.e., chronic endometriosis [19], dysfunctional endometrial bleeding [20], endometrial polyps [21], endometrial cancer [22], and preterm delivery [17]. The uterus of women with endometrial cancer is harbored by *Atopobium vaginae*, *Acinetobacter, Cloacibacterium*, *Comamonadaceae*, *Escherichia*, *Porphyromonas*, and *Pseudomonas* [23,24]. In women suffering from vaginitis, an overgrowth of *Bacteroides*, *Mobiluncus*, *Trichomonas vaginalis*, and *Candida* species was seen in vaginal smears [25]. The dysbiosis of different sites of the reproductive tract was not only related by the emergence of reproductive diseases but it can also disrupt reproductive functions, leading to infertility. In this context, women who had microbial species *Propionibacterium*, *Streptococcus*, *Actinomyces*, *Bifidobacterium*, and *Propionibacterium* in their follicular fluid had less embryo transfer and pregnancy outcomes following in vitro fertilization and embryo transfer [26]. In women of reproductive age, it has been reported that about 30% to 71% of women suffering infertility showed endometriosis. Furthermore, implantation failure and pregnancy loss are associated with the negative effects of endometrial microbiota that are not dominated by *Lactobacillus* [27,28].

In women, reproductive tract microbial biodiversity is highly dynamic and affected by several factors, mainly hormonal changes related to the stage of the reproductive cycle. High estrogen levels, such as around ovulation time, have been found to contribute to the maintenance of reproductive tract eubiosis. Estrogens maintain the acidity of the vaginal fluids, which makes them suitable media for the growth of beneficial bacteria lactobacilli. On the other hand, low levels of estrogens and the increment in pH such as that induced around menstrual period can shift the reproductive tract from eubiosis to dysbiosis through lowering the number of lactobacilli and increasing the opportunity of undesirable microorganisms’ growth, increasing the susceptibility of infectious pathogens [29,30]. 

In men, the disbiosis of the male reproductive tract’s microbiome leads to several inflammatory diseases (epididymitis, orchitis, and prostatitis), subfertility, and poor semen quality [31]. For example, the higher frequency of *Mycoplasma hominis* was seen in infertile men than in fertile men [32]. An increase in *Neisseria*, *Pseudomonas*, and *Klebsiella* accompanied by a reduction of *Lactobacillus* has recently been linked to oligoasthenoteratozoospermia and seminal hyper viscosity [33]. In another study, the seminal plasma microbiome of chronic prostatitis men contained a higher population of the inflammatory *Proteobacteria* and a lesser population of the friendly bacteria Lactobacilli compared to those of healthy men [34].

### 2.2. Animal Model

The relationship between the reproductive tract microbiota biodiversity and reproductive health has also been reported in animals. The dominance of certain genera in the vaginal microbiota in female bovine have been linked to reproductive disorders. For instance, increased relative abundance of *Bacteroides* and *Enterobacteriaceae* have been shown in cows with reproductive disease compared to healthy cows [35]. In bovine, the abundance of *Bacteroidetes* and decreased *Parvimonas*, *Porphyromonas*, unclassified *Veillonellaceae*, and *Mycoplasma* was associated with bovine necrotic vulvovaginitis [36]. The prevalence of cervical *Bacteroidetes* and *Fusobacteria* was associated with metritis [37]. In sheep, the presence of *Campylobacter* in vaginal microbiota was associated with increased rates of abortion [38].

## 3. Manipulation of Reproductive Tract Microbiota

These findings highlight the role of reproductive tract eubiosis in achieving adequate reproductive health and fertility and the importance of fixing any dysbiosis that might be raised due to unpleasant factors.

The manipulation of the microbiota existing in the reproductive tract of both males and females can be an effective tool to control many reproductive diseases and associated infertility. Reproductive outcomes in both humans [39,40] and animal models [14,41] may be improved by decreasing the proportion of dysbiotic microorganisms and microbial pathogens in the reproductive tract and increasing the proportion of symbiotic microorganisms. According to this concept, the use of antibiotics and the use of probiotics are basically two approaches that may be used to manage reproductive tract microbiota. In this context, several studies have confirmed the beneficial effects of antibiotics to tackle many reproductive microbial infectious diseases and related infertility. Antibiotics-based treatment of patients with chronic endometriosis has been found to improve live birth rate from 7% to 56% [42,43], the pregnancy and live birth rates [44], and the implantation and pregnancy rates of patients with recurrent implantation failure either with or without chronic endometriosis [45]. Prophylactic antibiotics therapy prior to oocyte retrieval in patients suffering severe endometriosis has also been reported with an infection risk reaching 0% [2,46]. Despite these benefits, on the other hand, treatment with antibiotics, even if for the short-term, can negatively affect the fertility of males and females. For example, evidences from rodent and amphibian models confirm that short-term antibiotics treatment (cefmetazole, ceftazidime ciprofloxacin, doxycycline, enrofloxacin, gentamycin, ofloxacin, salinomycin, streptomycin, and tetracycline) can impair spermatogenesis, decrease sperm motility, suppress androgen production by Leydig cells, and generate oxygen-free radicals within the male reproductive tract [3]. Currently, a treatment of male mice with different antibiotics (clindamycin, Unasyn: ampicillin/sulbactam, and Baytril: enrofloxacin), even for a short-term period (14 days), resulted in metabolome alterations at the seminal fluid (inosine, xanthine, and l-glutamic acid) and histopathological changes at the epididymides (cribriform growth and mitotic figures; [4]).

The negative effects of antibiotics on reproductive organs and functions can be related to the lack of the specificity of broad-spectrum antibiotics, which could not only impair the growth of dysbiotic bacteria but also symbiotic bacteria. Furthermore, the widespread use of antibiotics poses a serious health risk due to the development of multiple-antibiotic-resistant microbial species. Furthermore, antibiotics-based treatments are linked to high rates of recurrent infections after treatment and side effects derived from the clearance of naturally harbored flora in other body sites [5]. These aspects evoke scientists to find safer alternatives for maintaining reproductive system eubiosis, which can be probiotics.

## 4. Probiotics and Reproductive Health

### 4.1. Definition and Characteristics

The term ‘probiotics’ has come to be used to refer to the live microorganisms that, when administered in adequate amounts, confer a health benefit to the host [47]. More recent attention has focused on the possible roles of probiotics in restoring the reproductive tract eubiosis, while restricting antibiotics use to minimal levels [48,49]. In the field of reproductive medicine, *Lactobacillus* species (*Lactobacillus reuteri* RC-14, *Lactobacillus fermentum*, *Lactobacillus gasseri*, *Lactobacillus rhamnosus*, *Lactobacillus acidophilus*, *Lactobacillus crispatus*, *Lactobacillus casei*, and *Lactobacillus salivarius*), *Bifidobacterium* species, and *Bacillus* species are the major representatives of probiotics for modulating fertility dysbiosis [40]. The properties and functions of some probiotic strains used to improve reproductive functions and reproductive health are shown in Table 1.

Probiotic microbial species should have certain criteria to be approved as being safe compounds for health and effective when they are used for prophylactic and/or therapeutic interventions. In this term, safety properties (hemolytic activity, antibiotic resistance, and allergic and inflammatory activities) and functional properties (acid, bile salt, and lysozyme tolerance; gastrointestinal survival; antagonistic activity against pathogens; hydrophobicity; auto-aggregation and co-aggregation abilities; biofilm formation; adhesion capacity to reproductive tract and digestive systems epithelial cells; biosynthesis of active antimicrobial molecules) should be tested before approving any microorganism as a potential probiotic [5,50]. These properties determine to a high degree the efficiency of microbial strains to be used as probiotics. Among safety properties, the absence of antibiotic resistance genes has to be confirmed to avoid the possibility of a horizontal transfer of antibiotic resistance genes to pathogens and reproductive tract commensal microbiota, worsening the problem of antibiotic resistance [5]. In this regard, [60] suggested that yeast-based probiotics may be safer potential candidates because not only are they naturally resistant to antibiotics, and so it is not necessary to evaluate their antibiotic resistance profile, but also because they can be used in patients subjecting to antibiotic therapy without negative effects on the efficiency of probiotics-based therapy [60]. 

Functional properties of probiotics are also important to achieve effective probiotics-based therapy. The properties such as tolerance to acids, bile salts, and lysozyme and gastrointestinal survival are related to the ability of these microorganisms to survive through the gastrointestinal system and to transfer to other target sites in body such as reproductive organs. Thus, these properties may be very important for oral-based administration to ensure the transfer of the probiotics to other sites of action, conferring local and direct effects on target organs. On the other hand, adhesion capacity may be more important when probiotics are vaginally administrated to allow a direct and targeted colonization action of the probiotics for restoring unhealthy vaginal microbiota [40,62]. In fact, the colonization rate of the cervicovaginal communities after one cycle of probiotic treatment depends on several factors such as probiotic species, administration route (oral or vaginal), and other individual-dependent variables [52,63,64,65]. Dhanasekar and co-workers [6] reported that the strains *Lactobacillus rhamnosus* GR1 and *Lactobacillus Reuteri* RC14 may persist up to 19 days in the human vagina following intravaginal administration. Both strains survive at low pH, have high adherent ability to uroepithelial and vaginal cells, and are able to colonize the vagina when administered orally, whereas some strains such as *Lactobacillus rhamnosus* GG and *Lactobacillus acidophilus* are not well-suited to colonize the vagina or to produce H_2_O_2_ (has antimicrobial activity), explaining why both these strains fail to prevent the recurrence of urogenital infections [6]. 

### 4.2. Potential Sources

#### 4.2.1. Human Reproductive Tract

According to the previous facts on the eligibility criteria of microorganisms to act as an effective probiotic strain for maintaining and restoring reproductive health and functioning, it is supposed that one important potential source of probiotic strains is the reproductive tract itself [28]. Innovating products that contain probiotic strains of reproductive tract origin may increase their successful adaptation and colonization in the reproductive tract environment relative to probiotic strains isolated from other origins [52]. Pino and co-workers [50] assessed *Lactobacilli* isolated from the vaginas of healthy women for their potential safety and functional properties. Of 119 assessed strains, 7 identified strains (*Lactobacillus rhamnosus*, E21 and L3, *Lactobacillus helveticus*, P7, P12, S7, and U13, and *Lactobacillus salivarius*, N30) have been found to typically fulfill most criteria of probiotics. In the same study, 6 out of 119 strains belonging to *Lactobacillus crispatus* and *Lactobacillus gassseri* species have been found to exhibit phenotypical resistance to vancomycin. 

#### 4.2.2. Animal Reproductive Tract

El-Deeb and co-workers [66] isolated 18 Lactobacillus species from the vaginas of dromedary camels, (belonging to *Lactobacillus plantarum*, *fermentum* and *rhamnosus*) *with potential probiotic properties.* These bacterial species also exhibit phenotypical resistance to vancomycin-resistant strains and sensitivity to streptomycin, erythromycin, kanamycin, and chloramphenicol [66]. In fact, however, this criterion may raise some fear toward the antimicrobial resistance issue; the different mechanism of antimicrobial resistance of these strains compared to other bacteria such as *Enterococci* may preclude this fear, as the antimicrobial resistance of these strains is attributed to the synthesis of a modified cell-wall peptidoglycan precursor and is different from the transferable mechanism observed for other bacteria [50].

The probiotic properties of microbial species isolated from different sites of the reproductive tract has been confirmed in many studies. Chenoll and co-workers [52] confirmed the protective role of the vaginal-isolated strain *Lactobacillus rhamnosus* BPL005 (CECT 8800) against the bacterial colonization of primary endometrial epithelial cells infected with *Atopobium vaginae*, *Gardnerella vaginalis*, *Propioni bacterium acnes*, and *Streptococcus agalactiae* pathogens. In the same context, *Lactobacillus crispatus* is the most frequent colonizing species in healthy women with antimicrobial activity, and it is therefore nominated to be used as a probiotic therapeutic alternative to antibiotics [67]. In another study, 4 of 47 lactic acid strains (LAB) isolated from the vagina of cows (LAB1, LAB3, LAB5, and LAB9) demonstrated antibacterial activity against *Weissella confusa*, *Enterococcus hirae*, *Leuconostoc lactis*, and *Ilyobacter polytropus*. However, these strains showed different hydrophobicity, antibacterial activity, and adherence ability. Thus, the authors suggested that the use of a mixture of these strains can be a more efficient intervention for developing probiotic products that can prevent and treat reproductive tract infections in cows. Similarly, Ametaj and co-workers [53] proved the efficiency of a combination of three lactic acid bacteria species, *Lactobacillus sakei* FUA 3089, *Pediococcus acidilactici* FUA 3138, and FUA 3140, isolated from the vaginal tract of healthy cows to decrease the incidence of metritis, pyometra, and vaginal purulent discharges, as well as the number of cows with abnormal cervical size and uterine fluctuation. In addition, vaginal isolates of dromedary camels (*Camelus dromedarius*) have been found to contain *Lactobacillus* species belonging to *Lactobacillus plantarum*, *Lactobacillus fermentum*, and *Lactobacillus rhamnosus* strains with *potential probiotic properties.* These bacterial species showed strong antimicrobial activity against *Staphylococcus aureus*, which is considered as one of the most common bacterial causes of endometritis in dromedary camels and other animal species [66]. 

Overall, these studies performed either on human or animal models confirm that the reproductive tract microbial communities represent a suitable source of probiotic candidates that could be used in new functional formulates for maintaining and restoring reproductive tract eubiosis.

### 4.3. Mechanisms of Action

Probiotics can maintain and restore reproductive tract eubiosis through different mechanisms. Probiotics have been found to produce bioactive molecules that have direct effects on reproductive tract microbiota, as these bioactive molecules can act as antimicrobial agents. In a pathogenic process, the microbiome shifts to a dysbiotic state, in which pH is increased to values above 4.5 and *Lactobacilli* drop, whilst there is an overgrowth of other groups such as *Gardnerella*, *Atopobium*, *Prevotella*, and *Streptococcus*, among others [68]. The protective capacity of many probiotic species is based on their ability to produce short-chain fatty acids such as acetic, propionic, and lactic acids. The dominance of acid-producing microbial strains, such as *Lactobacilli*, in the uterine lumen and vaginal microbiome help to maintain acidic milieu, which provides an efficient protective agent against pathogenic microorganisms [16].

Some probiotic species, in addition to the ability of many species to reduce the pH, are able to produce oxidative molecules such as hydrogen peroxide, as well as another group of molecules known as bacteriocins (peptidic toxins; [21,52,69]). Both hydrogen peroxide and bacteriocins have pronounced antimicrobial activity against microbial pathogens. Probiotics have been also found to improve the immune responses and inflammatory status of the reproductive tract. For instance, the administration of probiotics resulted in a reduction of endometritic lesions through improving endometrial epithelial cells barrier function, boosting the activity of natural killer cells and increasing interleukin-12 levels [1].

As shown in Table 1, some probiotic species exhibit antioxidant activity. This activity can be mediated by several mechanisms: (1) the metal ion chelating ability, specifically to Fe^2+^ or Cu^2+^; (2) the synthesis of their own antioxidant enzymes such as Fe- or Mn-superoxide dismutase and Catalase; (3) the production of metabolites with antioxidant activity, such as glutathione, butyrate, and folate; (4) controlling antioxidant signaling pathways (nuclear factor kappa-light-chain-enhancer of activated B cells: NFκB, *mitogen-activated protein kinase:* MAPK, and *protein kinase: C:* PKC); and (5) the regulation of the enzymes producing reactive oxygen species such as NADPH oxidase complex [70].

Other studies have highlighted another mechanism of action of probiotic that is mediated by the hypothalamic–pituitary–*adrenal* (HPA) axis. Some probiotic species such as *Bifidobacterium infantis* and *Lactobacillus rhamnosus* (JB-1) and *farciminis* have been found to exhibit anxiolytic effects and improve psychiatric-disorder-related behaviors, including anxiety, depression, acute stress disorders, and obsessive disorders [71,72]. These effects were developed through their influence on the HPA-axis [72], which can indirectly improve reproductive functions by attenuating negative effects of different environmental stresses on reproductive functions. Furthermore, some probiotic species can produce neurotransmitters such as dopamine, serotonin, and gamma-aminobutyric acid that interact with the nervous system [31].

### 4.4. Benefits of Probiotic for Female Reproduction

#### 4.4.1. Women’s Fertility

Among reproductive diseases in women, bacterial vaginosis represents one of the major pathogenic diseases in women of reproductive age. Bacterial vaginosis is caused by vaginal dysbiosis and predominance of pathogenic microorganisms (bacteria, fungi, and yeast). It is characterized by several clinical and uncomfortable symptoms such as the release of discharge with abnormal *vaginal odor*, burning with urination, and itching. Additionally, it is linked to the increased risk of early delivery (pre-term delivery) among pregnant women [6]. In many studies, probiotics have been successfully used as a treatment for bacterial vaginosis [12,13]; in this sense, women undergoing bacterial vaginosis and receiving *Lactobacillus* vaginal tablets revealed the disappearance of bacterial vaginosis symptoms and had a clinically normal vaginal microbiota after daily probiotic therapy application for just 7 days. Furthermore, long-term vaginal administration of *Lactobacillus rhamnosus* appears to be a useful, safe, and effective antibiotic complementary approach in the management of bacterial vaginosis [73]. In addition, the administration of *Lactobacillus rhamnosus GR1* and *reuteri RC14* in pregnant women restores the normal vaginal flora and acidic pH and interrupts the infectious/inflammatory process due to bacterial vaginosis with recovery rates comparable to those of tocolytic therapies. Thus, probiotics are preferred over tocolytic therapy to reduce the adverse maternal and fetal outcomes [6]. Probiotics have been confirmed to tackle other reproductive diseases, even those that are not microbial-pathogen-borne diseases. For example, Zhang and co-workers [45] found that oral treatment with *Bifidobacterium lactis* V9 was an effective treatment for tackling polycystic ovary syndrome, a hormonal-disturbance-induced disease. As hypothesized by the authors of this study, the effect of *Bifidobacterium lactis* V9 is mediated by the gut–brain axis, as *Bifidobacterium lactis* V9 produces certain amounts of short-chain fatty acids, which consequently increase the release of ghrelin and peptide YY. These latter two molecules can restore the hormonal profile of sex hormones, mainly LH and prolactin, at the brain level by a manner similar to normal hormonal profiles.

Interestingly, probiotics can be safely used during pregnancy and breastfeeding to offer a unique opportunity to influence a range of important maternal and neonatal outcomes. The oral supplementation with the probiotic *Lactobacillus rhamnosus* HN001 (6 × 10^9^ CFU/day) to women during early pregnancy and during the first 6 months of breastfeeding can reduce the rates of infant eczema and atopic sensitization at 1-year and maternal gestational diabetes mellitus, bacterial vaginosis, and depression and anxiety postpartum [74]. The findings of the effects of probiotic treatment on women’s fertility are shown in Table 2.

#### 4.4.2. Animal Fertility

The positive effects of probiotics on female reproduction are not only observed in humans but also in animal models. Specifically, farm animals may be of particular importance, as maintaining their health, improving reproductive and productive performance, and eliminating drugs/antibiotics use represent crucial challenges. Considering these aspects, many probiotic microbial species have been tested either for treating reproductive diseases or for improving the productivity of healthy farm animals (Table 2).

For example, the management of the estrous cycle in some farm animals (i.e., ruminants) includes many protocols that depend on intravaginal progesterone devices and/or sponges. In many cases, the administration of these intravaginal means has been found to generate histological and cytological alterations (hyperplasia, hypertrophy, hemorrhage and perivascular infiltrate, numbers and activities of phagocytes) at the time of its withdrawal, which evoke inflammatory responses and provoke abnormal vaginal discharges, impacting animal welfare aspects and subsequent reproductive performance [80]. In a recent study by Quereda and co-workers [10], a vaginal infusion of a combination of probiotic *Lactobacillus* species (60% *Lactobacillus crispatus*, 20% *Lactobacillus brevis*, and 20% *Lactobacillus gasseri*) at the time of the insertion of fluorogestone acetate (FGA) sponges for estrus synchronization in ewes restored normal vaginal microflora and improved fertility (60% vs. 91%; *p* = 0.097). 

In addition, probiotics-based treatments can be used to improve the postpartum health and reproductive performance of farm animals. Specifically, parturition and uterine involution during the postpartum period may expose females to many reproductive infectious diseases, mainly due to the associated disturbance in the metabolism and the increase in inflammatory responses [14,77,78,81]. In this context, postpartum cows treated in weeks −2 and −1 prepartum and in week 1 postpartum with an intravaginal combination of *Lactobacillus sakei* FUA3089, *Pediococcus acidilactici* FUA3138, and *Pediococcus acidilactici* FUA3140 had smaller cross-sectional areas of gravid horn and uterine body on day 14 postpartum, earlier uterine involution, and resumed ovarian cyclicity earlier. Generally, the positive effects of probiotic treatments on reproductive performance and cows’ health might be ascribed to the ability of *Lactobacillus* species to decrease postpartum uterine infection and thus to decrease endotoxin translocation into the systemic circulation, decreasing the occurrence of inflammatory responses [77,78]. 

Focusing on improving the productivity of animals, in sows, the addition of dietary *Clostridium butyricum* (0.1, 0.2, or 0.4%) from day 90 of gestation to weaning at Day 21 postpartum could shorten the duration of farrowing and enhance the growth performance of suckling piglets. Moreover, 0.2% *Clostridium butyricum* administration to sows changed the composition of intestinal microbiota, specifically increasing the relative abundance of *Prevotella* [41]. In addition, *Bacillus* species serve as safe and effective feed additives which can improve the reproductive performance of farm animals [82,83]. Gu and co-workers [11] found that dietary supplementation with *Bacillus subtilis* and/or *Bacillus licheniformis* improved the lipid and protein metabolism profiles of sows, the placental antioxidant capacity, and placental efficiency that resulted in greater piglet birth weight. In sheep, the administration of monensin or *Saccharomyces cerevisiae* improved the metabolic status and ovarian steroid production of dams, as well as the growth performance of lambs, highlighting the possibility of using probiotic *Saccharomyces cerevisiae* as an alternative to the antibiotic monensin [76]. The findings of the effects of probiotic treatment on female animals’ fertility are shown in Table 2.

### 4.5. Benefits of Probiotics for Male Reproduction

#### 4.5.1. Men’s Fertility

A summary of some studies on the effects of probiotics on men’s reproductive health and fertility is shown in Table 3. These studies have supported the use of probiotics-based therapy as a potential tool to enhance reproductive efficiency and to tackle infertility in males. Probiotics have been demonstrated to improve spermatogenesis, epididymal sperm count, and normal sperm percentage and to reduce the percentage of sperm morphological abnormalities and DNA damage in mammalian species, including humans [39].

These positive effects have been seen in different infertility-related models. Infertility related to age [87], obesity [54], prostate diseases [34], and psychological and environmental stresses [84], as well as idiopathic infertility [8], can be improved by administering probiotics-based therapies either alone [8,85] or as a co-therapy [9]. In fact, regardless the different causes, male infertility seems to be concomitant to the inadequacy of testosterone levels and other sex hormones and the increase in inflammatory responses and oxidative stress [39,84]. As reported in many studies, probiotics can improve the fertility of males mainly by improving testosterone levels, attenuating inflammatory responses, and reversing the adverse effects of oxidative stress [87].

#### 4.5.2. Animal Fertility

Given the fact that the number of studies on the effect of probiotics on men’s fertility is relatively low, animal model studies can provide guidance evidence and allow understanding the effect of probiotics in men’s fertility with different clinical states. For example, in a study by Ommati and co-workers [88], the consumption of 200 g/1000 mL water D-fructose (to induced non-alcoholic fatty liver disease, NAFLD model) by pubertal rats, simultaneously with a combination of Iranian yogurt-extracted/cultured probiotics (1  ×  10^9^ CFU/mL of *Lactobacillus acidophilus*, *Bifidobacterium* spp., *Bacillus coagulans*, and *Lactobacillus rhamnosus*), for 63 consecutive days, reversed all negative effects of NAFLD on semen quality and redox homeostasis in rats. Other studies (Table 3) have shown the ability of different probiotic strains to ameliorate negative impacts of some stresses such as toxicity, aging, and obesity on semen quality and fertility [54,84,89].

It is important to refer to the fact the administration of probiotics in males is mainly through the oral route; none of the published studies performed in males have shown another route of administration. This fact highlights the importance of a gut–testicular axis as a potential pathway mediating the effects of probiotics on male reproduction [87]. Interestingly, to the authors’ best knowledge, conversely to female studies, the effects of probiotics administration on the male reproductive tract microbiota have not been yet fully studied. This observation highlights the importance of directing more studies towards the understanding of the role of probiotic in restoring and modulating the microbiome in male reproductive tract and seminal plasma and its possibilities for tackling pathogenic diseases in males.

## 5. Safety and Hazards of Probiotic and Prospects

The current evidence of the positive effects of probiotics on reproductive health and fertility of different mammalian species make them a suitable tool for reproduction and increasing reproductive efficiency. Recently, a number of probiotics-based products, either hygienic or therapeutic products, are available, including, in addition to probiotic supplements, feminine hygiene products such as sanitary napkins, tampons, and panty liners [90]. Hence, further high-quality designed studies are still needed to confirm their safety, prior to recommending their systematic use to treat pathogenic microbial diseases (e.g., vaginosis, metritis, endometritis, and prostatitis), as well as other gynecological disorders such as preterm births. Currently, most probiotics are generally recognized as safe (GRAS) to health [91] not only for adults but also for neonates whose mothers were exposed during pregnancy or breastfeeding [92]. However, very few studies have reported a transient decrease in hemoglobin levels in children supplemented with probiotics before and after birth [93]. Accordingly, the wide distribution of such treatments still needs adequate and robust evaluations to ensure their health safety for treated individuals/animals, including the evaluation of the antibiotic resistance of adopted probiotic species to avoid possible development of antibiotic-resistant pathogens [94]. In addition, the potential of probiotic strains to produce toxin and hemolytic and hydrolytic enzymes (hemolysin, gelatinase, lecithinase, etc.) should be tested. Moreover, more details in terms of identifying efficient probiotic species, route of administration, length and time of administration, and mechanisms of action are required to consider probiotics-based treatments as an approved pharmaceutical therapy or as a co-therapy [62,95]. To ensure all these aspects, each eligible probiotic strain must be tested following the four stages of drug development, including discovery and development, preclinical study, clinical study, and approval by the Food and Drug Administration (FDA). As an advanced step towards the commercialization of probiotic-based products, the FDA has included such products under a new “live biotherapeutic products” (LBP) category, which helps guarantee the quality, safety, and efficacy of new LBP products through following the FDA regulations [96].

The candidate probiotic species, in addition to health safety as discussed above, should have functional and industrial properties, enabling them to be stable during pharmaceutical processing and storage and to survive afterwards in different biological environments, such as the gastrointestinal and reproductive tracts. In this context, the emergence of some recent industrial technologies such as micro/nanoencapsulation technology can help to improve the stability of probiotics [94]. The emerging studies in this field showed the efficiency of some nanocarriers such as polyvinylalcohol nanofibers to maintain the viability of vaginal administrated probiotic products for a longer time, allowing the colonization of the introduced probiotic strains for a longer time in the reproductive tract [97]. Another strategy to improve the functional and industrial properties of probiotic strains includes encompassing prebiotics (nutrients that support probiotic strains growth) such as inulin (a rich fructan-type oligosaccharide supports the growth of *Lactobacillus* and *Bifidobacterium*) [98] and mushroom extract [99] in the formula. 

Currently, the most recent research trends refer to the possibility of the use of postbiotics (metabolic and bioactive products secreted by probiotics, such as enzymes, proteins, amino acids, peptides, organic acids, vitamins, and bacteriocins) as an alternative to probiotics itself. The use of postbiotics might have offered several advantages over probiotics, such as obtaining a specific mechanism of action and biological role, an easier production process for industrial-scale-up, and, lastly, a better availability and accessibility during marketing and application [100].

## 6. Conclusions

The dysbiosis of the reproductive tract microbiota due to the action of pathogens and/or unhealthy lifestyle has been related to many reproductive diseases and disorders in both humans and animal models. Recent studies have focused on the possible roles of different probiotics in restoring the reproductive tract eubiosis. Among the different candidate species, *Lactobacillus, Bifidobacterium*, and *Bacillus* are the most adopted for modulating reproductive tract dysbiosis [40]. The biological activities of probiotics (including antioxidant, antimicrobial, anti-inflammatory, and immunomodulatory activities) provide promising opportunities to maintain and restore reproductive eubiosis and to improve reproductive performance and fertility traits [52]. A highly significant issue is that the use of probiotics in offering an opportunity to decrease the use of antibiotics. However, great efforts should be paid to confirm the lack of antibiotic resistance of the probiotics themselves to avoid the vertical transfer of antibiotics-resistant genes to pathogens or other microorganisms normally harbored by the host [50,52]. 

Hence, future studies have to focus on identifying mechanisms by which probiotics can affect reproductive processes, the safety of probiotics to the host specifically when they are consumed during sensitive reproductive windows such as pregnancy, and finally, the litigation and regulatory rules required for marketing these biological-based therapies with sufficient safety and hazards instructions [100].

## Figures and Tables

**Table 1:** Safety and functional properties of some probiotics used to improve reproductive functions and reproductive health.

Probiotic Species (Reference)	Properties/Function
*Lactobacillus rhamnosus* (E21 and L3) *Lactobacillus helveticus* (P7, P12, S7, and U13) *Lactobacillus salivarius* (N30) [50]	➢ High survival during in vitro gastrointestinal passage ➢ Adhesion to both intestinal and vaginal epithelia ➢ Hydrophobicity ➢ Auto-aggregation ➢ Co-aggregation ➢ Reduce pH ➢ Produce organic acids (mainly acetic acid) and hydrogen peroxide (H_2_O_2_) ➢ Inhibit Candida species growth
*Lactobacillus* strain (SQ0048) [51]	➢ Colonizes the vaginal microflora of healthy cows ➢ Serves as a significant microbiological barrier to genital pathogen infections ➢ Has adhering ability to the specific epithelium ➢ Produces bacteriocins
*Lactobacillus reuteri* RC14 *Lactobacillus rhamnosus* GR1 [6]	➢ Excellent colonizing ability ➢ Preferred thereby of urogenital tract infections ➢ Tolerate low pH ➢ High adherent ability to uroepithelial and vaginal cells ➢ Colonizes the vagina when is administered orally ➢ Integral part of female genital tract
*Lactobacillus rhamnosus* BPL005 [52]	➢ Reduces PH ➢ Produces organic acids (mainly acetic acid) ➢ Suppress *Propionibacterium acnes* and *Streptococcus agalactiae* growth ➢ No signs of cytotoxicity, irritation in vaginal, or allergic contact dermatitis potential
*Lactobacillus buchneri* (DSM 32407) [14,53]	➢ Does not affect viability of epithelial cells ➢ Dose not evoke pro-inflammatory response ➢ Improves antioxidant status ➢ Reduces PH ➢ Produces organic acids such as lactic acid ➢ Produces H_2_O_2_ and bacteriocins ➢ Produces co-aggregation molecules that block the spread of pathogens
*Lactobacillus reuteri* ATCC PTA 6475 [54]	➢ Anti-inflammatory strain
*Lactobacillus rhamnosus CICC6141* *Lactobacillus casei BL23v* [55]	➢ Has adhering ability to the epithelial gut capacities
*Lactobacillus rhamnosus* CECT8361 *Bifidobacterium longum* CECT7347 [56]	➢ Hs antioxidant and anti-inflammatory activities
*Lactobacillus gasseri OLL2809* [57]	➢ Has immunostimulatory activity
*Bacillus amyloliquefaciens* [58]	➢ Tolerates high temperature ➢ Reduces pH ➢ Improves antioxidant status
*Bacillus subtilis* [11] *Lactobacillus rhamnosus* CECT8361 and *Bifidobacterium longum* CECT7347 [56]	➢ Have antioxidant activity
*Bacillus subtilis* (DSM10) *Bacillus clausii* (DSM 8716) *Bacillus coagulans* (DSM 1) *Bacillus amyloliquefaciens* (DSM 7) [59]	➢ Safe *Bacillus* species with probiotic properties
*Bifidobacterium lactis* V9 [45]	➢ Probiotic characteristics
*Saccharomyces cerevisiae* [60,61]	➢ Induces pathogen co-aggregation ➢ Has antibiotic resistance profile ➢ Has anti-inflammatory properties ➢ Suppresses *Candida albicans* growth

**Table 2:** Summary of some studies on the effects of probiotics on female reproductive health and fertility.

Specie (References)	Probiotic Treatment	Results
	Studies on Women	
Women [6]	*Lactobacillus rhamnosus* GR1 and *Lactobacillus reuteri* RC14	➢ Restored the normal vaginal flora and acidic pH ➢ Decreased preterm delivery
Pregnant women [7]	Tablets containing *Bifidobacterium longum* (5 × 10^6^ CFU), *Lactobacillus delbrueckii bulgaricus* (5 × 10^5^ CFU), and *Streptococcus thermophilus* (5 × 10^5^ CFU) Two tablets twice a day from week 32 of pregnancy to delivery	➢ Helped to shift the anti-inflammatory state to a pro-inflammatory state in the third trimester, which is important for labor
Women [45]	*Bifidobacterium lactis* V9	➢ Treatment of polycystic ovary syndrome
Women with vulvovaginal candidiasis [75]	Oral intake of *Saccharomyces cerevisiae* CNCM I-3856 (5 × 10^9^ CFU/mL) Once a day for 56 days	➢ Decreased candida vaginal load
Women [74]	*Lactobacillus rhamnosus* HN001(6 × 10^9^ CFU/day) From 14−16 days of pregnancy to 6 months of breastfeeding	➢ Reduced the rates of infant eczema and atopic sensitization at 1 year ➢ Decreased maternal gestational diabetes mellitus, bacterial vaginosis, and depression and anxiety postpartum
Women [12]	*Lactobacillus*-containing vaginal tablets	➢ Restored a normal vaginal microbiota in patient with bacterial vaginosis
	Studies on animals	
Ewes [10]	A combination of 60% *Lactobacillus crispatus*, 20% *Lactobacillus brevis,* and 20% *Lactobacillus gasseri* at fluorogestone acetate sponge insertion	➢ Reduced vaginal smears neutrophils count ➢ Reduced *Enterobacteriaceae* counts ➢ Increased vaginal microbial diversity ➢ Improved fertility rate
Late pregnant sows [11]	Different combinations of 0.5% isomaltooligosaccharide (IMO), 0.02% *Bacillus subtilis*, and 0.02% *Bacillus licheniformis*	➢ Improved metabolism of sows, the placental antioxidant capacity, umbilical venous serum growth hormone concentrations ➢ Improved piglet birth weight
Landrace × Yorkshire sows [41]	Basal diets supplemented with 0, 0.1%, 0.2%, or 0.4% *Clostridium butyricum* (4 × 10^8^ CFU/kg) From day 90 of gestation to weaning at day 21 of lactation	➢ Shortened the duration of farrowing ➢ Enhanced the growth performance of suckling piglets ➢ 0.2% Clostridium *butyricum* increased the relative abundance of *Prevotella*
Ghezel ewes [76]	Dietary supplementation with 30 mg/ewe/day of monensin (MS) or 4 × 10^9^ of CFU/ewe/d *Saccharomyces cerevisiae* (SC)	➢ MS and SC treatments resulted in: ➢ Greater number and heavier lambs ➢ Increased concentrations of estradiol, progesterone, blood urea nitrogen, insulin, glucose, cholesterol, and total protein
Cows with signs of subclinical endometritis [14]	Intrauterine administration of the *Lactobacillus buchneri* DSM 32407	➢ Stimulated local immune system function ➢ Downregulated the endometrial mRNA expression of several pro-inflammatory factors ➢ Improved the reproductive performance of cows with subclinical endometritis
Cows [77]	A combination of *Lactobacillus* rhamnosus, *Pediococcus acidilactici*, and *Lactobacillus reuteri* at a ratio of 25:25:2	➢ Reduced *Escherichia coli* infection in uterus explants ➢ Reduced acute inflammation caused by *Escherichia coli*
Dairy cows [78]	A combination of *Lactobacillus sakei* FUA3089, *Pediococcus acidilactici* FUA3138, and *Pediococcus acidilactici* FUA3140 (10^8^−10^9^ CFU/dose) Weekly from 2 weeks prepartum to 1 week postpartum	➢ Decreased NEFA ➢ Increased milk IgG ➢ Increased milk efficiency of transitioning dairy cows
Late pregnant sows [79]	Dietary *Bacillus*	➢ Improved piglet birth weight ➢ Reduced the rate of stillbirths and number of weak piglets
Late pregnant cows [53]	Intravaginal administration of *Lactobacillus sakei* FUA 3089, *Pediococcus acidilactici* FUA 3140, and *Pediococcus acidilactici* FUA 3138 (10^10^–10^12^ CFU/cow) Weekly from two weeks prepartum to 4 weeks postpartum	➢ Decreased incidence of purulent vaginal discharges ➢ Decreased plasma haptoglobin, an acute phase protein often associated with uterine infections ➢ Enhanced milk production of cows

CFU: Colony-forming units.

**Table 3:** Summary of some studies on the effects of probiotics on male reproductive health and fertility.

Specie (References)	Probiotic Treatment	Results
Studies on men		
Idiopathic asthenozoospermia men [8]	Tablets containing *Lactobacillus casei*, *Lactobacillus rhamnosus*, *Lactobacillus bulgaricus*, *Lactobacillus acidophilus*, *Bifidobacterium breve*, *Bifidobacterium longum*, and *Streptococcus thermophil* (2 × 10^11^ CFU)	➢ Improved serum testosterone, ejaculate volume, sperm concentration, percentage of motile sperm, and total antioxidant capacity of plasma ➢ Decreased concentration of plasma malondialdehyde and inflammatory markers
Men with idiopathic oligoasthenoteratospermia [9] et al., 2017	A combination of *Lactobacillus paracasei* B21060 (5 × 10^9^ cells) + arabinogalctan (1243 mg) + oligo-fructosaccharides (700 mg) + L-glutamine (500 mg) for 6 months	➢ Improved sperm concentration, ejaculate volume, progressive motility normal morphological sperm cells, and sex hormone levels (follicle stimulating hormones, luteinizing hormone, and testosterone)
Asthenozoospermic men [56]	*Lactobacillus rhamnosus* CECT8361 and *Bifidobacterium longum* CECT7347 for six weeks	➢ Improved sperm motility ➢ Reduced DNA fragmentation and intracellular H_2_O_2_
Studies on animals		
Stressed mice [84]	*Lactobacillus rhamnosus* Gorbach–Goldin An oral dose of 0.3 mL/mouse (1 × 10^10^ cells/mL)	➢ Improved semen quality and testosterone levels ➢ Decreased inflammatory response (cyclooxygenase 2, IL-1β, IL-6, and tumor necrosis factor-α) and oxidative stress (malondialdehyde and protein carbonyls)
Brioler [58]	Dietary supplementation with *Bacillus* *amyloliquefaciens*	➢ Increased sperm concentration and percentage of live sperm cells, and antioxidant activity
Mice [85]	*Lactobacillus rhamnosus* PB01 (DSM 14870) supplementation	➢ Improved sperm kinematic properties
Aging and/or obese mice [54]	*Lactobacillus reuteri* ATCC PTA 6475 (3.5 × 10^5^ cell/mouse/day)	➢ Increased spermatogenesis, Leydig cell numbers, serum testosterone ➢ Downregulating systemic pro-inflammatoryIL-17A-dependent signaling
Mice exposed to cyclophosphamide toxicity [86]	Dietary administration of *Lactobacillus casei* (10^5^, 10^6^, 10^7^, 10^8^ CFU/g) for 8 weeks	➢ Increased testicular and epididymal weights, sperm concentrations, percentages of morphological normality, and serum superoxide dismutase and testosterone levels.

CFU: Colony-forming units.

## Data Availability

Not applicable.

## References

[1] Molina N.M., Sola-Leyva A., Saez-Lara M.J., Plaza-Diaz J., Tubic-Pavlovic A., Romero B., Clavero A., Mozas-Moreno J., Fontes J., Altmae S. (2020). New Opportunities for Endometrial Health by Modifying Uterine Microbial Composition: Present or Future. Biomolecules.

[2] Pereira N., Hutchinson A.P., Lekovich J.P., Hobeika E., Elias R.T. (2016). Antibiotic prophylaxis for gynecologic procedures prior to and during the utilization of assisted reproductive technologies: A systematic review. J. Pathog..

[3] Silla A.J., Keogh L.M., Byrne P.G. (2015). Antibiotics and oxygen availability affect the short-term storage of spermatozoa from the critically endangered booroolong frog, Litoria booroolongensis. Reprod. Fertil. Dev..

[4] Rosenfeld C.S., Javurek A.B., Johnson S.A., Lei Z., Sumner L.W., Hess R.A. (2018). Seminal fluid metabolome and epididymal changes after antibiotic treatment in mice. Reproduction.

[5] Moreno I., Simon C. (2019). Deciphering the effect of reproductive tract microbiota on human reproduction. Reprod. Med. Biol..

[6] Dhanasekar K.R., Shilpa B., Gomathy N., Kundavi S. (2019). Prenatal Probiotics: The Way Forward in Prevention of Preterm Birth. J. Clin. Gynecol. Obstet..

[7] Chen Y., Li Z., Tye K.D., Luo H., Tang X., Liao Y., Wang D., Zhou J., Yang P., Li Y. (2019). Probiotic Supplementation During Human Pregnancy Affects the Gut Microbiota and Immune Status. Front. Cell. Infect. Microbiol..

[8] Helli B., Kavianpour M., Ghaedi E., Dadfar M., Haghighian H.K. (2020). Probiotic effects on sperm parameters, oxidative stress index, inflammatory factors and sex hormones in infertile men. Hum. Fertil..

[9] Maretti C., Cavallini G. (2017). The association of a probiotic with a prebiotic (Flortec, Bracco) to improve the quality/quantity of spermatozoa in infertile patients with idiopathic oligoasthenoteratospermia: A pilot study. Andrology.

[10] Quereda J.J., Garcia-Rosello E., Barba M., Moce M.L., Gomis J., Jimenez-Trigos E., Bataller E., Martinez-Bovi R., Garcia-Munoz A., Gomez-Martin A. (2020). Use of Probiotics in Intravaginal Sponges in Sheep: A Pilot Study. Animals.

[11] Gu X.L., Li H., Song Z.H., Ding Y.N., He X., Fan Z.Y. (2019). Effects of isomaltooligosaccharide and Bacillus supplementation on sow performance, serum metabolites, and serum and placental oxidative status. Anim. Reprod. Sci..

[12] Mastromarino P., Macchia S., Meggiorini L., Trinchieri V., Mosca L., Perluigi M., Midulla C. (2009). Effectiveness of Lactobacillus-containing vaginal tablets in the treatment of symptomatic bacterial vaginosis. Clin. Microbiol. Infect..

[13] Bradshaw C.S., Pirotta M., De Guingand D., Hocking J.S., Morton A.N., Garland S.M., Fehler G., Morrow A., Walker S., Vodstrcil L.A. (2012). Efficacy of oral metronidazole with vaginal clindamycin or vaginal probiotic for bacterial vaginosis: Randomised placebo-controlled double-blind trial. PLoS ONE.

[14] Peter S., Gartner M.A., Michel G., Ibrahim M., Klopfleisch R., Lubke-Becker A., Jung M., Einspanier R., Gabler C. (2018). Influence of intrauterine administration of Lactobacillus buchneri on reproductive performance and pro-inflammatory endometrial mRNA expression of cows with subclinical endometritis. Sci. Rep..

[15] Younge N., McCann J.R., Ballard J., Plunkett C., Akhtar S., Araújo-Pérez F., Murtha A., Brandon D., Seed P.C. (2019). Fetal exposure to the maternal microbiota in humans and mice. JCI Insight.

[16] Sheldon I.M., Cronin J.G., Healey G.D., Gabler C., Heuwieser W., Streyl D., Bromfield J.J., Miyamoto A., Fergani C., Dobson H. (2014). Innate immunity and inflammation of the bovine female reproductive tract in health and disease. Reproduction.

[17] Wee B.A., Thomas M., Sweeney E.L., Frentiu F.D., Samios M., Ravel J., Gajer P., Myers G., Timms P., Allan J.A. (2018). A retrospective pilot study to determine whether the reproductive tract microbiota differs between women with a history of infertility and fertile women. Aust. N. Z. J. Obstet. Gynaecol..

[18] Herath S., Williams E.J., Lilly S.T., Gilbert R.O., Dobson H., Bryant C.E., Sheldon I.M. (2007). Ovarian follicular cells have innate immune capabilities that modulate their endocrine function. Reproduction.

[19] Moreno I., Franasiak J.M. (2017). Endometrial microbiota—New player in town. Fertil. Steril..

[20] Pelzer E.S., Willner D., Buttini M., Huygens F. (2018). A role for the endometrial microbiome in dysfunctional menstrual bleeding. Antonie Van Leeuwenhoek.

[21] Fang R.-L., Chen L.-X., Shu W.-S., Yao S.-Z., Wang S.-W., Chen Y.-Q. (2016). Barcoded sequencing reveals diverse intrauterine microbiomes in patients suffering with endometrial polyps. Am. J. Transl. Res..

[22] Rizzo A.E., Gordon J.C., Berard A.R., Burgener A.D., Avril S. (2021). The Female Reproductive Tract Microbiome—Implications for Gynecologic Cancers and Personalized Medicine. J. Pers. Med..

[23] Walther-António M.R., Chen J., Multinu F., Hokenstad A., Distad T.J., Cheek E.H., Keeney G.L., Creedon D.J., Nelson H., Mariani A. (2016). Potential contribution of the uterine microbiome in the development of endometrial cancer. Genome Med..

[24] Walsh D.M., Hokenstad A.N., Chen J., Sung J., Jenkins G.D., Chia N., Nelson H., Mariani A., Walther-Antonio M.R. (2019). Postmenopause as a key factor in the composition of the Endometrial Cancer Microbiome (ECbiome). Sci. Rep..

[25] Koedooder R., Mackens S., Budding A., Fares D., Blockeel C., Laven J., Schoenmakers S. (2019). Identification and evaluation of the microbiome in the female and male reproductive tracts. Hum. Reprod. Update.

[26] Pelzer E.S., Allan J.A., Waterhouse M.A., Ross T., Beagley K.W., Knox C.L. (2013). Microorganisms within human follicular fluid: Effects on IVF. PLoS ONE.

[27] Halis G., Arici A. (2004). Endometriosis and inflammation in infertility. Ann. N. Y. Acad. Sci..

[28] López-Moreno A., Aguilera M. (2021). Vaginal probiotics for reproductive health and related dysbiosis: Systematic review and meta-analysis. J. Clin. Med..

[29] Fuochi V., Li Volti G., Furneri P.M. (2017). Commentary: Lactobacilli dominance and vaginal pH: Why is the human vaginal microbiome unique?. Front. Microbiol..

[30] Russo R., Karadja E., De Seta F. (2019). Evidence-based mixture containing Lactobacillus strains and lactoferrin to prevent recurrent bacterial vaginosis: A double blind, placebo controlled, randomised clinical trial. Benef. Microbes.

[31] Altmäe S., Franasiak J.M., Mändar R. (2019). The seminal microbiome in health and disease. Nat. Rev. Urol..

[32] Ahmadi M.H., Mirsalehian A., Gilani M.A.S., Bahador A., Talebi M. (2017). Asymptomatic infection with Mycoplasma hominis negatively affects semen parameters and leads to male infertility as confirmed by improved semen parameters after antibiotic treatment. Urology.

[33] Monteiro C., Marques P.I., Cavadas B., Damião I., Almeida V., Barros N., Barros A., Carvalho F., Gomes S., Seixas S. (2018). Characterization of microbiota in male infertility cases uncovers differences in seminal hyperviscosity and oligoasthenoteratozoospermia possibly correlated with increased prevalence of infectious bacteria. Am. J. Reprod. Immunol..

[34] Mändar R., Punab M., Borovkova N., Lapp E., Kiiker R., Korrovits P., Metspalu A., Krjutškov K., Nolvak H., Preem J.-K. (2015). Complementary seminovaginal microbiome in couples. Res. Microbiol..

[35] Rodrigues N., Kästle J., Coutinho T., Amorim A., Campos G., Santos V., Marques L., Timenetsky J., De Farias S. (2015). Qualitative analysis of the vaginal microbiota of healthy cattle and cattle with genital-tract. Gen. Mol. Res..

[36] Shpigel N., Adler-Ashkenazy L., Scheinin S., Goshen T., Arazi A., Pasternak Z., Gottlieb Y. (2017). Characterization and identification of microbial communities in bovine necrotic vulvovaginitis. Vet. J..

[37] Wang Y., Wang J., Li H., Fu K., Pang B., Yang Y., Liu Y., Tian W., Cao R. (2018). Characterization of the cervical bacterial community in dairy cows with metritis and during different physiological phases. Theriogenology.

[38] Saleh M., Haraki Nezhad M.T., Salmani V. (2014). Detection of some bacterial causes of abortion in Afshari sheep using real time PCR detection and sensitivity assessment of campylobacter primers. Agric. Biotechnol. J..

[39] Younis N., Mahasneh A. (2020). Probiotics and the envisaged role in treating human infertility. Middle East Fertil. Soc. J..

[40] Lopez-Moreno A., Aguilera M. (2020). Probiotics Dietary Supplementation for Modulating Endocrine and Fertility Microbiota Dysbiosis. Nutrients.

[41] Cao M., Li Y., Wu Q.J., Zhang P., Li W.T., Mao Z.Y., Wu D.M., Jiang X.M., Zhuo Y., Fang Z.F. (2019). Effects of dietary Clostridium butyricum addition to sows in late gestation and lactation on reproductive performance and intestinal microbiota. J. Anim. Sci..

[42] Kitaya K., Matsubayashi H., Takaya Y., Nishiyama R., Yamaguchi K., Takeuchi T., Ishikawa T. (2017). Live birth rate following oral antibiotic treatment for chronic endometritis in infertile women with repeated implantation failure. Am. J. Reprod. Immunol..

[43] McQueen D.B., Bernardi L.A., Stephenson M.D. (2014). Chronic endometritis in women with recurrent early pregnancy loss and/or fetal demise. Fertil. Steril..

[44] Cicinelli E., Matteo M., Trojano G., Mitola P.C., Tinelli R., Vitagliano A., Crupano F.M., Lepera A., Miragliotta G., Resta L. (2018). Chronic endometritis in patients with unexplained infertility: Prevalence and effects of antibiotic treatment on spontaneous conception. Am. J. Reprod. Immunol..

[45] Zhang Y., Xu H., Liu Y., Zheng S., Zhao W., Wu D., Lei L., Chen G. (2019). Confirmation of chronic endometritis in repeated implantation failure and success outcome in IVF-ET after intrauterine delivery of the combined administration of antibiotic and dexamethasone. Am. J. Reprod. Immunol..

[46] Weinreb E.B., Cholst I.N., Ledger W.J., Danis R.B., Rosenwaks Z. (2010). Should all oocyte donors receive prophylactic antibiotics for retrieval?. Fertil. Steril..

[47] Hill C., Guarner F., Reid G., Gibson G.R., Merenstein D.J., Pot B., Morelli L., Canani R.B., Flint H.J., Salminen S. (2014). Expert consensus document: The International Scientific Association for Probiotics and Prebiotics consensus statement on the scope and appropriate use of the term probiotic. Nat. Rev. Gastroenterol. Hepatol..

[48] Khalesi S., Bellissimo N., Vandelanotte C., Williams S., Stanley D., Irwin C. (2019). A review of probiotic supplementation in healthy adults: Helpful or hype?. Eur. J. Clin. Nutr..

[49] Trush E.A., Poluektova E.A., Beniashvilli A.G., Shifrin O.S., Poluektov Y.M., Ivashkin V.T. (2020). The evolution of human probiotics: Challenges and prospects. Probiotics Antimicrob. Proteins.

[50] Pino A., Bartolo E., Caggia C., Cianci A., Randazzo C.L. (2019). Detection of vaginal lactobacilli as probiotic candidates. Sci. Rep..

[51] Niu C., Cheng C., Liu Y., Huang S., Fu Y., Li P. (2019). Transcriptome profiling analysis of bovine vaginal epithelial cell response to an isolated lactobacillus strain. Msystems.

[52] Chenoll E., Moreno I., Sanchez M., Garcia-Grau I., Silva A., Gonzalez-Monfort M., Genoves S., Vilella F., Seco-Durban C., Simon C. (2019). Selection of New Probiotics for Endometrial Health. Front. Cell. Infect. Microbiol..

[53] Ametaj B., Iqbal S., Selami F., Odhiambo J., Wang Y., Gänzle M., Dunn S., Zebeli Q. (2014). Intravaginal administration of lactic acid bacteria modulated the incidence of purulent vaginal discharges, plasma haptoglobin concentrations, and milk production in dairy cows. Res. Vet. Sci..

[54] Poutahidis T., Springer A., Levkovich T., Qi P., Varian B.J., Lakritz J.R., Ibrahim Y.M., Chatzigiagkos A., Alm E.J., Erdman S.E. (2014). Probiotic microbes sustain youthful serum testosterone levels and testicular size in aging mice. PLoS ONE.

[55] Zhou Z., Wang W., Liu W., Gatlin III D.M., Zhang Y., Yao B., Ringø E. (2012). Identification of highly-adhesive gut Lactobacillus strains in zebrafish (Danio rerio) by partial rpoB gene sequence analysis. Aquaculture.

[56] Valcarce D., Genovés S., Riesco M., Martorell P., Herráez M., Ramón D., Robles V. (2017). Probiotic administration improves sperm quality in asthenozoospermic human donors. Benef. Microbes.

[57] Sashihara T., Sueki N., Furuichi K., Ikegami S. (2007). Effect of growth conditions of Lactobacillus gasseri OLL2809 on the immunostimulatory activity for production of interleukin-12 (p70) by murine splenocytes. Int. J. Food Microbiol..

[58] Inatomi T., Otomaru K. (2018). Effect of dietary probiotics on the semen traits and antioxidative activity of male broiler breeders. Sci. Rep..

[59] Jeżewska-Frąckowiak J., Seroczyńska K., Banaszczyk J., Jedrzejczak G., Żylicz-Stachula A., Skowron P.M. (2018). The promises and risks of probiotic Bacillus species. Acta Biochim. Pol..

[60] Gaziano R., Sabbatini S., Roselletti E., Perito S., Monari C. (2020). Saccharomyces cerevisiae-Based Probiotics as Novel Antimicrobial Agents to Prevent and Treat Vaginal Infections. Front. Microbiol..

[61] Pericolini E., Gabrielli E., Ballet N., Sabbatini S., Roselletti E., Cayzeele Decherf A., Pélerin F., Luciano E., Perito S., Jüsten P. (2017). Therapeutic activity of a Saccharomyces cerevisiae-based probiotic and inactivated whole yeast on vaginal candidiasis. Virulence.

[62] Buggio L., Somigliana E., Borghi A., Vercellini P. (2019). Probiotics and vaginal microecology: Fact or fancy?. BMC Womens Health.

[63] Bohbot J., Cardot J. (2012). Vaginal impact of the oral administration of total freeze-dried culture of LCR 35 in healthy women. Infect. Dis. Obstet. Gynecol..

[64] Antonio M.A., Meyn L.A., Murray P.J., Busse B., Hillier S.L. (2009). Vaginal colonization by probiotic Lactobacillus crispatus CTV-05 is decreased by sexual activity and endogenous Lactobacilli. J. Infect. Dis..

[65] Hemmerling A., Harrison W., Schroeder A., Park J., Korn A., Shiboski S., Foster-Rosales A., Cohen C.R. (2010). Phase 2a study assessing colonization efficiency, safety, and acceptability of Lactobacillus crispatus CTV-05 in women with bacterial vaginosis. Sex. Transm. Dis..

[66] El-Deeb W.M., Fayez M., Elsohaby I., Ghoneim I., Al-Marri T., Kandeel M., ElGioushy M. (2020). Isolation and characterization of vaginal Lactobacillus spp. in dromedary camels (Camelus dromedarius): In vitro evaluation of probiotic potential of selected isolates. PeerJ.

[67] Kim J.-M., Park Y.J. (2017). Probiotics in the prevention and treatment of postmenopausal vaginal infections. J. Menopausal Med..

[68] Srinivasan S., Hoffman N.G., Morgan M.T., Matsen F.A., Fiedler T.L., Hall R.W., Ross F.J., McCoy C.O., Bumgarner R., Marrazzo J.M. (2012). Bacterial communities in women with bacterial vaginosis: High resolution phylogenetic analyses reveal relationships of microbiota to clinical criteria. PLoS ONE.

[69] Coudeyras S., Jugie G., Vermerie M., Forestier C. (2008). Adhesion of human probiotic Lactobacillus rhamnosus to cervical and vaginal cells and interaction with vaginosis-associated pathogens. Infect. Dis. Obstet. Gynecol..

[70] Wang Y., Wu Y., Wang Y., Xu H., Mei X., Yu D., Wang Y., Li W. (2017). Antioxidant properties of probiotic bacteria. Nutrients.

[71] Cani P.D., Knauf C. (2016). How gut microbes talk to organs: The role of endocrine and nervous routes. Mol. Metab..

[72] Sudo N., Chida Y., Aiba Y., Sonoda J., Oyama N., Yu X.N., Kubo C., Koga Y. (2004). Postnatal microbial colonization programs the hypothalamic–pituitary–adrenal system for stress response in mice. J. Physiol..

[73] Marcone V., Calzolari E., Bertini M. (2008). Effectiveness of vaginal administration of Lactobacillus rhamnosus following conventional metronidazole therapy: How to lower the rate of bacterial vaginosis recurrences. New Microbiol..

[74] Barthow C., Wickens K., Stanley T., Mitchell E.A., Maude R., Abels P., Purdie G., Murphy R., Stone P., Kang J. (2016). The Probiotics in Pregnancy Study (PiP Study): Rationale and design of a double-blind randomised controlled trial to improve maternal health during pregnancy and prevent infant eczema and allergy. BMC Pregnancy Childbirth.

[75] Cayzeele-Decherf A., Pélerin F., Jüsten P. (2017). Saccharomyces cerevisiae CNCM I-3856 as a natural breakthrough for vaginal health: A clinical study. Med. J. Obstet. Gynecol..

[76] Ahmadzadeh L., Hosseinkhani A., Kia H.D. (2018). Effect of supplementing a diet with monensin sodium and Saccharomyces Cerevisiae on reproductive performance of Ghezel ewes. Anim. Reprod. Sci..

[77] Genis S., Sanchez-Chardi A., Bach A., Fabregas F., Aris A. (2017). A combination of lactic acid bacteria regulates Escherichia coli infection and inflammation of the bovine endometrium. J. Dairy Sci..

[78] Deng Q., Odhiambo J., Farooq U., Lam T., Dunn S., Ametaj B. (2016). Intravaginal probiotics modulated metabolic status and improved milk production and composition of transition dairy cows. J. Anim. Sci..

[79] Kritas S., Marubashi T., Filioussis G., Petridou E., Christodoulopoulos G., Burriel A., Tzivara A., Theodoridis A., Pískoriková M. (2015). Reproductive performance of sows was improved by administration of a sporing bacillary probiotic (Bacillus subtilis C-3102). J. Anim. Sci..

[80] Hashem N.M., Gonzalez-Bulnes A. (2021). Nanotechnology and Reproductive Management of Farm Animals: Challenges and Advances. Animals.

[81] Deng F., McClure M., Rorie R., Wang X., Chai J., Wei X., Lai S., Zhao J. (2019). The vaginal and fecal microbiomes are related to pregnancy status in beef heifers. J. Anim. Sci. Biotechnol..

[82] Mizubuchi H., Yajima T., Aoi N., Tomita T., Yoshikai Y. (2005). Isomalto-oligosaccharides polarize Th1-like responses in intestinal and systemic immunity in mice. J. Nutr..

[83] Mao K., Liu L., Mo T., Pan H., Liu H. (2015). Preparation, characterization, and antioxidant activity of an isomaltooligosaccharide–iron complex (IIC). J. Carbohydr. Chem..

[84] Guo Y., Du X., Bian Y., Wang S. (2020). Chronic unpredictable stress-induced reproductive deficits were prevented by probiotics. Reprod. Biol..

[85] Dardmeh F., Alipour H., Gazerani P., Van der Horst G., Brandsborg E., Nielsen H.I. (2017). Lactobacillus rhamnosus PB01 (DSM 14870) supplementation affects markers of sperm kinematic parameters in a diet-induced obesity mice model. PLoS ONE.

[86] Yang J., Wen M., Pan K., Shen K., Yang R., Liu Z. (2012). Protective effects of dietary co-administration of probiotic Lactobacillus casei on CP-induced reproductive dysfunction in adult male Kunming mice. Vet. Res..

[87] Tremellen K., Pearce K. (2017). Probiotics to improve testicular function (Andrology 5: 439–444, 2017)—A comment on mechanism of action and therapeutic potential of probiotics beyond reproduction. Andrology.

[88] Ommati M.M., Li H., Jamshidzadeh A., Khoshghadam F., Retana-Márquez S., Lu Y., Farshad O., Nategh Ahmadi M.H., Gholami A., Heidari R. (2022). The crucial role of oxidative stress in non-alcoholic fatty liver disease-induced male reproductive toxicity: The ameliorative effects of Iranian indigenous probiotics. Naunyn-Schmiedebergs Arch. Pharmacol..

[89] Javurek A.B., Spollen W.G., Johnson S.A., Bivens N.J., Bromert K.H., Givan S.A., Rosenfeld C.S. (2017). Consumption of a high-fat diet alters the seminal fluid and gut microbiomes in male mice. Reprod. Fertil. Dev..

[90] Nader-Macías M.E.F., De Gregorio P.R., Silva J.A. (2021). Probiotic lactobacilli in formulas and hygiene products for the health of the urogenital tract. Pharmacol. Res. Perspect..

[91] Mattia A., Merker R. (2008). Regulation of probiotic substances as ingredients in foods: Premarket approval or “generally recognized as safe” notification. Clin. Infect. Dis..

[92] Baldassarre M.E., Palladino V., Amoruso A., Pindinelli S., Mastromarino P., Fanelli M., Di Mauro A., Laforgia N. (2018). Rationale of Probiotic Supplementation during Pregnancy and Neonatal Period. Nutrients.

[93] Kuitunen M., Kukkonen K., Savilahti E. (2009). Pro-and prebiotic supplementation induces a transient reduction in hemoglobin concentration in infants. J. Pediatr. Gastroenterol. Nutr..

[94] Czarnecki-Maulden G.L. (2008). Effect of dietary modulation of intestinal microbiota on reproduction and early growth. Theriogenology.

[95] Hoffmann D.E., Fraser C.M., Palumbo F., Ravel J., Rowthorn V., Schwartz J. (2014). Probiotics: Achieving a better regulatory fit. Food Drug Law J..

[96] Cordaillat-Simmons M., Rouanet A., Pot B. (2020). Live biotherapeutic products: The importance of a defined regulatory framework. Exp. Mol. Med..

[97] Nagy Z.K., Wagner I., Suhajda Á., Tobak T., Harasztos A.H., Vigh T., Sóti P.L., Pataki H., Molnár K., Marosi G. (2014). Nanofibrous solid dosage form of living bacteria prepared by electrospinning. eXPRESS Polym. Lett..

[98] Li T., Zhang Y., Song J., Chen L., Du M., Mao X. (2022). Yogurt Enriched with Inulin Ameliorated Reproductive Functions and Regulated Gut Microbiota in Dehydroepiandrosterone-Induced Polycystic Ovary Syndrome Mice. Nutrients.

[99] Zakariaee H., Sudagar M., Hosseini S.S., Paknejad H., Baruah K. (2021). In vitro Selection of Synbiotics and in vivo Investigation of Growth Indices, Reproduction Performance, Survival, and Ovarian Cyp19α Gene Expression in Zebrafish Danio rerio. Front. Microbiol..

[100] Nataraj B.H., Ali S.A., Behare P.V., Yadav H. (2020). Postbiotics-parabiotics: The new horizons in microbial biotherapy and functional foods. Microb. Cell Factories.

